# Breeding potential of Spanish bread wheat landraces: genetic variability in vernalization and photoperiod sensitivity

**DOI:** 10.3389/fpls.2025.1593667

**Published:** 2025-10-30

**Authors:** Estela Giménez, Laura Pascual, Matilde López-Fernández, José G. Vázquez-García, Marta García-Mondejar, Magdalena Ruiz, Patricia Giraldo

**Affiliations:** ^1^ School of Agricultural, Food and Biosystems Engineering, Department of Biotechnology-Plant Biology, Universidad Politécnica de Madrid, Madrid, Spain; ^2^ Instituto Nacional de Investigación y Tecnología Agraria y Alimentaria (INIA), CSIC, Finca La Canaleja, Alcalá de Henares, Madrid, Spain

**Keywords:** Triticum aestivum, vernalization, photoperiod, wheat landraces, growth habit, phenology

## Abstract

Flowering time and grain filling, regulated by temperature-responsive (vernalization) and day length (photoperiod) genes, are critical factors in wheat production. In this study, we analyzed genetic variation at the *VRN-A1, VRN-B1, PPD-A1, PPD-B1, PPD-D1, EPS-A1, EPS-D1*, and *WAPO-A1* loci in 188 Spanish wheat landraces and compared them to 28 modern cultivars. Growth habit and phenological traits (days to heading and maturity) were assessed in field trials, and the impact of genetic variability on these traits was evaluated. Our results revealed distinct genetic diversity patterns between landraces and modern cultivars and confirmed that the absence of spring alleles at *VRN1* loci was necessary for a winter growth habit. Dominant alleles from *VRN1* gene (*Vrn-A1a, Vrn-A1b, Vrn-B1a*) and photoperiod-insensitive alleles from *PPD1* gene (*Ppd-B1a, Ppd-D1a*) consistently promoted early heading and flowering, despite genotype-by-environment (G×E) interactions. Interestingly, *Ppd-D1b* allele, although classified as photoperiod-insensitive exhibited, delayed flowering likely due to its molecular variation. While allelic variation at *EPS1* locus had no significant impact on phenology, results suggested a potential role for *WAPO1* in flowering regulation. Our findings highlight the genetic diversity within Spanish wheat landraces, offering valuable genetic resources for optimizing flowering time and improving wheat adaptation to complex agro-climatic conditions.

## 1 Introduction

Wheat (*Triticum* sp.) is one of the most widely cultivated cereals used for human consumption. According to the Food and Agriculture Organization of the United Nations (FAO), to feed the projected 10 billion people by 2050, food production needs to increase by 70% without expanding the current area of arable land (FAO, 2009). The primary challenge for modern agriculture is achieving this goal in a scenario where climate projections indicate instability in rainfall patterns and a reduction in precipitation, all while not increasing, or even reducing, nitrogen fertilization levels. For wheat production, one key factor is the transition from vegetative to reproductive growth and grain filling. These processes are finely regulated by molecular mechanisms that respond to both endogenous and environmental stimuli, generating enormous plasticity in the flowering process to ensure that floral development only takes place under optimal environmental conditions (Hill and Li, 2016). The grain yield is directly affected by timing of flowering, as it must occur during an optimal season to minimize exposure to abiotic stresses (such as cold, heat and drought) and biotic stresses (including fungi, bacteria, viruses, nematodes, and insects). Regarding abiotic stresses, they affect yield risk primarily through alignment (or misalignment) between the wheat´s reproductive critical period and optimal environmental conditions. Flohr et al. (2017) emphasized that late flowering respect to optimal conditions can lead to damages by heat and drought while Dreccer et al. (2018) described that early flowering can lead to freeze damage. Wheat’s adaptability around the world is primarily due to genes that regulate flowering through responses to vernalization and photoperiod, ensuring that flowering occurs at the right time (Gomez et al., 2014). Thus, the broad adaptability of wheat to diverse geographies and sowing times is closely linked to the utilization of natural allelic variation in photoperiod sensitivity and vernalization response genes (Hill and Li, 2016).

Vernalization and photoperiod sensitivity are regulated by *VERNALIZATION (VRN)* and *PHOTOPERIOD (PPD)* genes respectively. Vernalization is mainly regulated by three homoeologous loci across the A, B, and D genomes: *VRN1* (Yan et al., 2003), *VRN2* (Yan et al., 2004b), *VRN3* (Yan et al., 2006). In addition, *VRN-D4*, identified on chromosome 5D, also contributes to the vernalization response (Kippes et al., 2015). Many wild relatives of wheat carry recessive vernalization alleles associated with the winter growth habit (Yan et al., 2004a; Chu et al., 2011) whereas spring wheat varieties, capable of flowering without exposure to prolonged period of low temperatures during the vegetative stage, are thought to have evolved from such winter-type ancestors (Gomez et al., 2014). After the vernalization requirement is met, the photoperiod response, primarily influenced by the *PPD* loci, governs, and adjusts flowering timing for specific environments (Fjellheim et al., 2014). Wild ancestral wheats were long-day photoperiod-sensitive plants, and the photoperiod insensitivity observed in many wheat cultivars results from mutations in *PPD* genes, leading to an early flowering phenotype under both short and long-day conditions (Thomas and Vince-Prue, 1997). In addition to these two major pathways, earliness per se (*EPS*), or “narrow-sense earliness,” is determined by a set of loci with typically small effects (Hill and Li, 2016) that influence flowering once vernalization and photoperiod requirements are satisfied (Zikhali and Wingen, 2016). The circadian cycle regulator ortholog *EARLY FLOWERING 3 (ELF3*) of Arabidopsis was described as a candidate for the *EARLINESS PER SE-D1 (EPS-D1*) locus in *Triticum aestivum* L (Zikhali and Griffiths, 2015). and its homolog *EPS-A^m^1* in *Triticum monococcum* L (Alvarez et al., 2016). Allelic variation at these loci can advance flowering, although the magnitude and direction of the effect are strongly environmental conditions-dependent; as a consequence of the interaction between alleles from *EPS* and *VRN* genes with temperature and *PPD* alleles with day length (Prieto et al., 2020). Although the effects of *EPS* alleles are generally small, they can still cause detectable variation in flowering time independently of the major *VRN* and *PPD* genes (Van Beem et al., 2005).

Several genes that modify heading time by regulating the expression of *VRN1* (Alvarez et al., 2023; Li and Dubcovsky, 2008; Li et al., 2015; Lv et al., 2014; Shaw et al., 2019; Turner et al., 2005; Yan et al., 2006) have also been identified as regulators of the spikelet number per spike (SNS). Loss-of-function mutations in flowering promoting genes *PPD1* (Ning et al., 2023; Shaw et al., 2013) and *FT1/VRN3* (Chen et al., 2022, Shimizu et al., 2020) result in later heading and significant increases in SNS, whereas mutations in the *ELF3/EPS-D1* flowering repressor result in early heading and reduced SNS (Alvarez et al., 2023). Three other genes (*CONSTANS LIKE5 (COL5*), *WAPO1*, and *LEAFY (LFY)*) have also been shown to affect SNS. *COL5* overexpression in transgenic plants was associated with increases in SNS, suggesting that this gene operates as a positive regulator of SNS (Zhang et al., 2022). This seems to be also the case for *WAPO1* and *LFY*, since null mutations in the two paralogs of these genes result in reductions in SNS (Kuzay et al., 2022; Paraiso et al., 2024). However, the *WAPO1* exact role in flowering time regulation remains unclear; while native alleles have not been reported to cause large changes in flowering time in field traits, transgenic expression delayed flowering and CRISPR an EMS mutants had not significant effects (Muqaddasi et al., 2019; Katz et al., 2022; Kuzay et al., 2022).

The semi-dwarf, lodging-resistant, high-yield wheat varieties developed by the International Maize and Wheat Improvement Center (CIMMYT) during the “Green Revolution” replaced the local varieties traditionally grown by farmers (Smale et al., 2002). Since then, CIMMYT wheat germplasm has influenced almost all wheat improvement programs worldwide, contributing to the selection and fixation of favorable alleles but also leading to a partial loss of genetic diversity in the crop (Niu et al., 2023). Despite the targeted use of synthetic hexaploids and landrace introgressions has partially re-expanded diversity in recent breeding cycles, the still reduced genetic variability can limit the crop’s ability to respond to new needs and increases its vulnerability to climate change or the emergence of new pests or diseases. The genetic diversity found in collections of local wheat varieties and wild relatives is considered the reservoir of the lost diversity (Skovmand et al., 2001; King et al., 2024).

Currently, the most extensive and oldest collection of traditional Spanish wheat varieties is preserved at the National Genetic Resources and sustainable agriculture Center (CRF-INIA-CSIC). The active collection consists of 3,722 accessions, of which 1,551 are Spanish varieties (http://www.inia.es/inventarionacional), including 522 local bread wheat varieties (*Triticum aestivum* ssp. *vulgare*). In recent years, genotypic and phenotypic characterization has been done on a collection composed of 189 bread wheat landraces, selected for their agro-climatic variability and since they represent most of the genetic diversity of the complete bread wheat collection (Pascual et al., 2020a). This subset has been genotyped and phenotyped for agromorphological and grain quality traits (López-Fernández et al., 2023), and its genetic diversity and population structure have been evaluated (Pascual et al., 2020a and b; López-Fernández et al., 2021, 2023). However, a thorough characterization of the genetic variability related to phenology has yet to be fully explored in this germplasm.

Characterization of the allelic variation for *VRN*, *PPD*, *EPS* and *WAPO* genes in wheat landraces and modern cultivars provide a foundation for understanding phenological diversity. While landraces may harbor novel alleles, they can also carry linked deleterious variation and undesirable traits that complicate their direct use in breeding programs. The main objective of this work is to provide a genetic characterization of the main genes involved in flowering and maturity time among a collection of Spanish landraces and wheat modern cultivars, in order to better understand how allelic diversity correlates with flowering behavior. Such knowledge will be valuable for assessing phenology alleles when specific accessions are identified as promising sources of adaptive traits, enabling breeders to make informed decisions about their use.

## 2 Methods

### 2.1 Plant material

We selected a panel of 216 bread wheat (*Triticum aestivum* ssp. vulgare) lines, including 188 homozygous lines derived from bread wheat landraces and 28 reference cultivars which comprised modern cultivars widely grown in Spain during the last 50 years (**Supplementary Table S1** and review in Pascual et al., 2020b). In previous research, homozygous lines were derived by collecting single bagged spikes from single selected plants during three generations. Genetic identity and lack of heterozygosity were confirmed by SDS-PAGE protein profile (Pascual et al., 2020a). In addition, the genetic structure of the landraces collection was assessed from DArTseq genotyping data (Pascual et al., 2020a).

Across three seasons, 2017-2018, 2018–2019 and 2020-2021, the two hundred and sixteen genotypes (landraces and modern cultivars) were sown in late autumn and harvested in early summer, ensuring the vernalization and photoperiod conditions needed to guarantee flowering. The experiment followed a complete random design without replicates in plots of four rows (1 m long) separated from each other by 30 cm. At the trials were conducted at the Agricultural Experimental Station of ‘Escuela Técnica Superior de Ingeniería Agronómica, Alimentaria y de Biosistemas’ (40.44° N, 3.73° W) of the ‘Universidad Politécnica de Madrid (UPM)’ in Madrid, Spain. Minimum and maximum temperatures and precipitation were daily recorded for a weather station close to the experimental fields and the mean temperature and monthly accumulated precipitation are represented in **Supplementary Figure S1** to compare seasons. Field management practices during the experiments were in accordance with the standard agronomic practices commonly used in the area.

### 2.2 Phenotyping for phenological development

Days to heading (DH) and days to maturity (DM) were recorded as the days from sowing when more than 50% of the main spikes within a plot had reached Zadoks stage 55 and 87, respectively. Sown in all seasons were performed in late autumn (15th November-15th December). After sown plants were observed once per week until March when plants begun to be observed three times per week until the last genotype reached maturity. From 216 varieties, DH data were recorded in the three seasons (2017-2018, 2018–2019 and 2020-2021) and DM in the 2018–2019 and 2020–2021 seasons. DH and DM data were also available from previous studies (López-Fernández et al., 2023). Growth habit was visually determined according to standard protocols of the OEVV, seeds from 216 varieties were sown at 30^th^ March in 2019 and 2021. Those varieties capable of developing ears in both seasons were classified as Spring, those that only developed ears in one season as facultative, and those unable to flower as winter.

### 2.3 Molecular markers selected for vernalization and photoperiod related genes

A set of molecular markers associated to *VRN1, PPD1, EPS1* and *WAPO1* genes that included PCR, KASP and CAPS markers was selected from the literature (**Table 1**).

**Table 1:** Molecular markers used for genotyping the collection of wheat landraces and modern cultivars.

Gene	Marker	Assay	Allele	Polymorphysm	Response expected	Reference
*VRN-A1*	Vrn-A1_9K0001	KASP	*vrn-A1*	–	Winter	Rasheed et al., 2016
			*Vrn-A1a*	Insertion	Spring	
	VrnA1_k2	PCR	*vrn-A1*	–	Winter	Yan et al., 2004a
			*Vrn-A1a*	Insertion	Spring	
	Vrn-A1b-Marq	KASP	*vrn-A1*	–	Winter	Rasheed et al., 2016
			*Vrn-A1b*	Deletion	Spring	
	VrnA1_new	KASP	*T allele*	–	Long	Rasheed et al., 2016
			*C allele*	SNP	Short	
	exon7_C/T-VrnA1	KASP	*T allele*	–	Late	Rasheed et al., 2016
			*C allele*	SNP	Early	
*VRN-B1*	Indel marker	PCR	*vrn-B1*	–	Winter	Fu et al., 2005
			*Vrn-B1a*	Deletion	Spring	
*PPD-A1*	GS100-1027IND	KASP	*ppd-A1*	–	Sensitive	Rasheed et al., 2016
			*Ppd-A1a*	Deletion	Insensitive	
*PPD-B1*	gene copies insertion	PCR	*ppd-B1*	–	Sensitive	Dıaz et al., 2012
			*Ppd-B1a*	Insertion	Insensitive	
*PPD-D1*	TaPpdDD001	KASP	*ppd-D1*	–	Sensitive	Rasheed et al., 2016
			*Ppd-D1a*	Insertion	Insensitive	
	TaPpdDD002	KASP	*ppd-D1*	–	Sensitive	Rasheed et al., 2016
			*Ppd-D1b*	Deletion	Insensitive	
	TaPpdD1_exon7InDel	PCR & sequencing	*ppd-D1*	–	Sensitive	This work
			*Ppd-D1b*	Deletion	Insensitive	
*EPS-A1*	SNP	CAP	*eps-A^m^1l*	–	Late	Alvarez et al., 2016
			*Eps-A^m^1e*	SNP	Early	
*EPS-D1*	Exon 4 SNP	KASP	*eps-D1*	–	Late	Zikhali et al., 2016
			*Eps-D1a*	SNP	Early	
*WAPO-A1*	F-box SNP	KASP	*wapo-A1_h3*	–	Low SNS	This work
			*Wapo-A1_h2*	SNP	High SNS	
	Promoter Del	PCR	*wapo-A1_h3*	–	Low SNS	Kuzay et al., 2021
			*wapo-A1_h1*	Deletion	Low SNS	

For *VRN1* loci, the KASP assay Vrn-A1_9K0001 (Rasheed et al, 2016) and the causal PCR marker VrnA1_k2 (Yan et al., 2004a) detect a duplication including the promoter region distinguishing between winter *vrn*-*A1* allele and spring type *Vrn-A1a* allele, whereas the KASP assay Vrn-A1b-Marq detects a 20-bp deletion in the 5’ UTR distinguishing between winter *vrn*-*A1* allele and spring type *Vrn-A1b* allele (Yan et al., 2004a; Rasheed et al, 2016). Additionally, the KASP assay VrnA1_new distinguished between the short winter allele C_allele (Jagger type) and the long winter allele T_allele (2174 type) at exon 4 of *VRN-A1* gene (Dıaz et al., 2012; Rasheed et al, 2016) and the KASP assay Exon7_C/T_Vrn-A1 was used to distinguish an early heading winter C_allele and a late winter T_allele at exon 7 of *VRN-A1* gene (Li et al., 2013; Dıaz et al., 2012; Rasheed et al, 2016). Deletion alleles in the intron-1 region of the *VRN-B1* gene affecting the vernalization response were identified by PCR as described in Fu et al. (2005) (**Table 1**).

At *PPD1* loci, the 1027 bp *Ppd-A1a* insensitive allele at *PPD-A1* was analyzed with KASP marker GS100-1027IND (Beales et al., 2007; Rasheed et al, 2016). The copy number variation at *PPD-B1* gene associated to photoperiod-insensitive allele *Ppd-B1a* was assessed by PCR according to Dıaz et al. (2012). Two KASP markers for *PPD-D1* photoperiod insensitivity alleles were also analyzed, TaPpdDD001 targeting a 2-kb InDel in the promoter region (*Ppd-D1a* allele), and TaPpdDD002 targeting a 5 bp InDel in exon 7 (*Ppd-D1b* allele) (Rasheed et al, 2016). Additionally, the *Ppd-D1b* allele was confirmed by PCR and sequencing.

At *EPS1* loci, the *Eps-Am1e* and *Eps-Am1l* alleles associated to early and late heading respectively, were distinguished by CAPS marker (Alvarez et al, 2016). Additionally, one KASP assay was used for the *EPS-D1* exon 4 SNP, which distinguishes the alleles for early T allele (*Eps-D1a*) and late C allele (*eps-D1*) heading (Zikhali et al., 2016).

Regarding *WAPO-A1* gene, the *wapo-A1_h1*, *Wapo-A1_h2*, and *wapo-A1_h3* haplotypes associated to high and low SNS were distinguished by a combination of KASP and PCR-based molecular markers. The PCR marker targets a unique 115-bp deletion in the promoter region of *WAPO-A1* (h1 haplotype) (Kuzay et al., 2022). A KASP marker was designed for the C47F polymorphism in the F-box that is unique to *WAPO-A1* (h2 haplotype) (Kuzay et al., 2022).

All genes, alleles and haplotypes have been named according to guidelines from Boden et al. (2023): recessive alleles (winter, late, etc.) are written in lowercase and italics (for example, *vrn-B*1), while dominant alleles (spring, short, etc.) are written in italics with the first letter in uppercase. The last letter of the allele name, “a”, “b”, etc. (for instance, *Vrn-B1a*), refers to the specific allele. In addition, haplotypes are named like alleles, but finished with a “h” (*Vrn-B1a_h1*). On the other hand, genes/loci are written in uppercase and italics (*VRN-B1*) while proteins/factors are written in uppercase, without italics (VRN-B1).

### 2.4 Genotyping

DNA was isolated from young leaves of a single plant using a standard cetyltrimethyl-ammonium bromide (CTAB) procedure. DNA integrity was analyzed mediating visualization in agarose gel (1%) and DNA quality and quantity were analyzed in a nanodrop. The whole collection was genotyped with the set of molecular markers that included PCR, KASP and CAPS markers (**Table 1**).

For PCR-based markers, reactions were performed with NZYTaq II 2x Green Master Mix (NZYtech), 2 μM of each primer and 50 ng of genomic DNA, in a final volume of 25 μl, using the following program: 5 min 94°C, 35 cycles of 30s 94°C, 30s 60°C and 90s 72°C, and a final step of 10 min 72°C in the Mastercycler^®^ nexus Eppendorf thermocycler. PCR products were visualized in agarose gel (1%).

To confirm the 5 bp deletion in exon 7 from *PPD-D1* gene associated to *Ppd-D1b* allele PCR was performed with ppdD1norstarf1 (5’GCTCATTTCATCAGCCTTGTCT3’) and ppdD1norstarr1 (5’ATGGTATGCTCAAGTGCTCAAC3’) primers. PCR products were purified using SephadexTM G50 columns and sequencing analysis were performed by the MACROGEN_Spain company to align sequences obtained with reference sequence of *PPD-D1* gene.

*VRN-A1* locus was genotyped by KASP technology at LGC Genomics (www.lgcgenomics.com). The obtained results were then manually curated using the software SNPviewer2 version 4.0.0.0 (www.lgcgenomics.com). The remaining KASP markers were set up in a lightcycler LC96, Roche, using KASP-TF V4.0 2X Master Mix, KASP by Design Primer Mix and 50 ng of genomic DNA; and the LGC predesigned protocol for KASP markers (Pre-incubation 95°C for 10 min., 10 amplification cycles of 94°C for 20s. and touchdown 61°C - 55°C for 60s., 27 amplification cycles of 94°C for 20s. and 55°C for 60s., and 20 amplification cycles of 39°C for 10s. and 37°C for 30s.).

### 2.5 Statistical analysis

All statistical analyses were performed using R v.4.1.3 (R Core Team, 2022) and the InfoStat statistical package (Di Rienzo et al., 2020). For each season, descriptive statistics (mean, standard deviation, and maximum and minimum values) of DH and DM were calculated separately for landraces and modern cultivars. Data were tested for normality and homeocedasticity were checked by Shapiro-Wilk and Levene test respectively (p-value < 0.01). Analyses of Variance (ANOVA) were carried out to identify the influence of the different genes, environments and the interaction among genes or genotypes by environment (P-value < 0.05). All the factors were considered fixed effects. The Proportion of Total Variation Explained (PVE) by the fixed effects was computed as the ratio of the effect sum of squares to the total sum of squares (%). When ANOVA was significant, a Duncan’s test (P-value < 0.05) was conducted to compare the means.

## 3 Results

### 3.1 Growth habit and phenology of landraces and modern cultivars

Landraces and modern cultivars were classified in winter or spring based on the results of the spring sown trial. Results showed that 113 (60.1%) out of 188 landraces were spring, while only 65 (39.9%) were winter. Similar percentages were observed in modern cultivars, in which 16 were spring (59.3%) and 11 were winter (40.7%) (See **Supplementary Table S1**). In addition, 9 landraces and 1 modern cultivar were classified as facultative. Due to the low representation of facultative modern cultivar and landraces these genotypes were not used in final analysis (See **Supplementary Table S1**).

**Figure 1:**
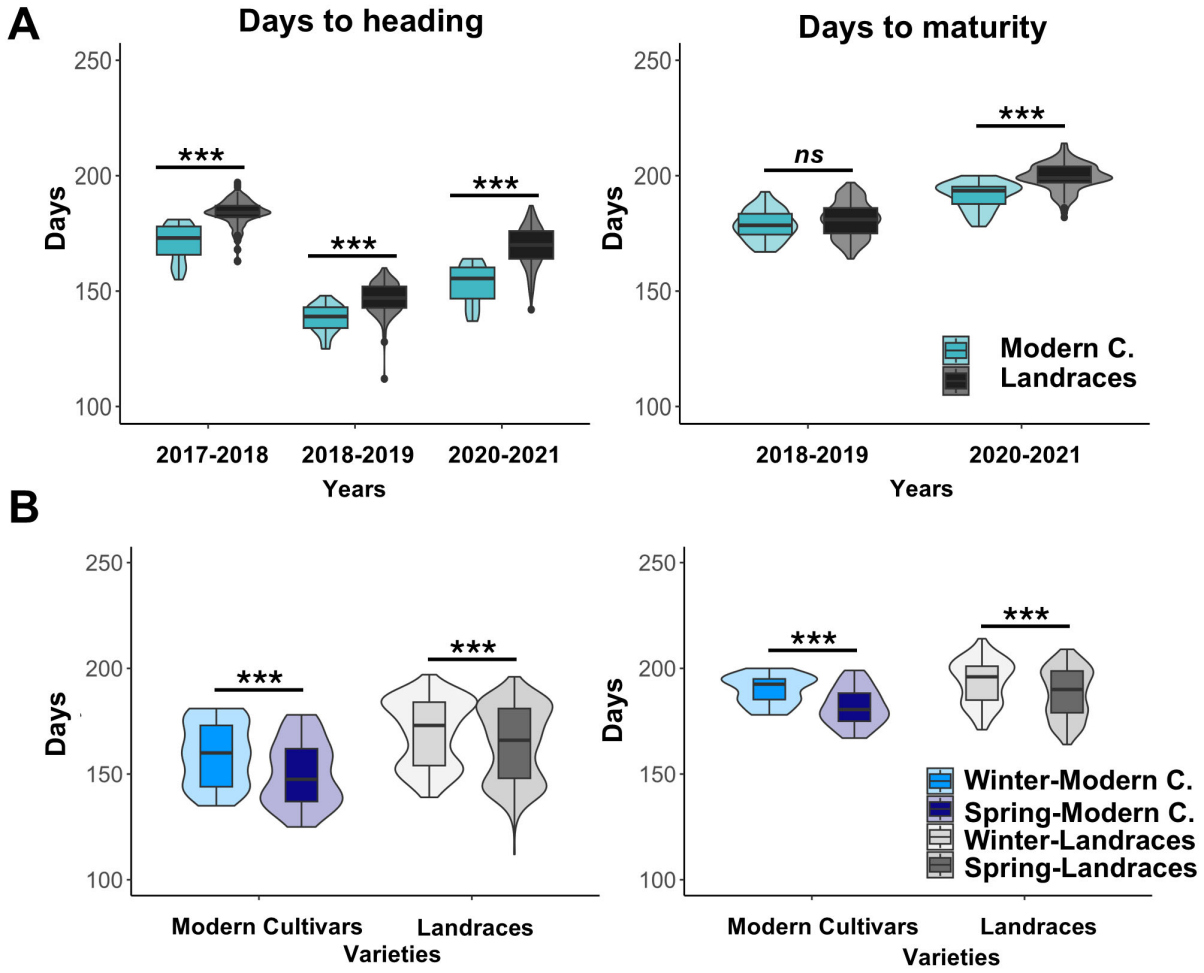
Days to heading and days to maturity in Spanish landraces and modern cultivars. **(A)** represent DH and DM values by seasons and type of material, **(B)** represent DH and DM values by growth habits. ns, not significant at p>0.05; *** indicate significance at p <0.0001.

Regarding crop development, the days from sowing to heading (DH) and from sowing to maturity (DM) stages were significantly higher in the landraces compared to the modern cultivars (**Figure 1A**). Across the 2017-2018, 2018–2019 and 2020–2021 seasons, DH mean values exhibited significant differences of 12, 8 and 16 days between both groups, respectively. Regarding to DM, these differences were 2 (non-significant) and 8 days (significant) in the 2018–2019 and 2020–2021 seasons, respectively (**Figure 1A**).

As expected, winter varieties showed higher DH and DM than spring varieties in both sets. Winter varieties required more time to reach heading with an average delay of about five days in both modern cultivars and landraces. Regard to reach maturity, winter varieties needed approximately 6 and 8 days more than spring varieties in landraces and modern cultivars, respectively. DH and DM differences observed in modern cultivars were significantly bigger than those observed in landraces (**Figure 1B**). These results are maintained across the three seasons, where winter varieties needed more days to reach DH and DM than spring. Differences in days are consistent across seasons except for difference of DH in 2017–2018 season in landraces, suggesting that the environment has a bigger effect on landraces varieties.

### 3.2 Genetic variability for *VRN1, PPD1, EPS1* and *WAPO1* genes in landraces and modern cultivars and relationship to growth habit

**Figure 2:**
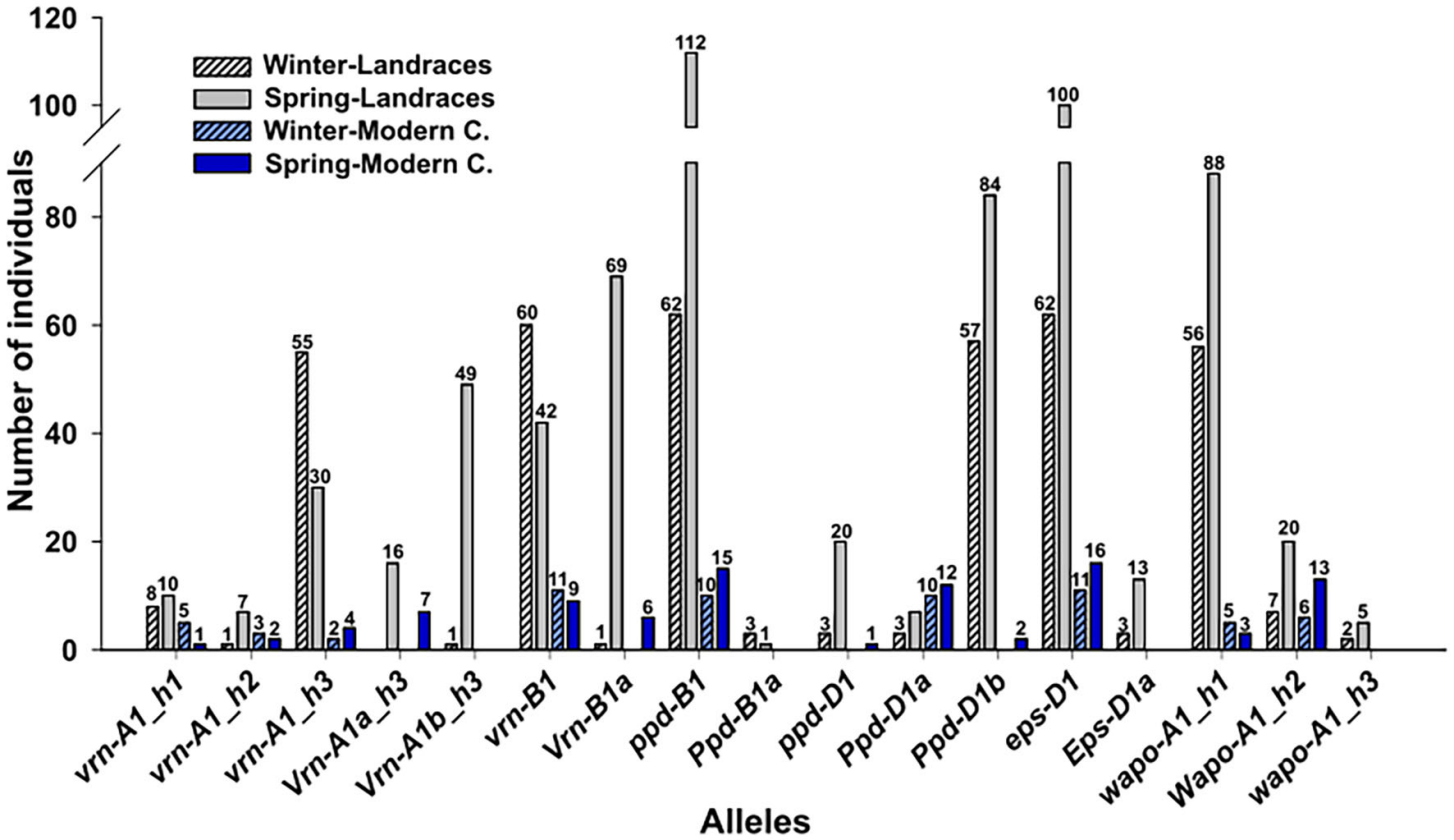
Allelic distribution of *VRN1*, *PPD1*, *EPS1* and *WAPO1* genes in landraces and modern cultivars. Numbers indicate the varieties included in each category, landraces are represented in grey and modern cultivars in blue. Winter lines are marked in dark color and spring type are dashed.

At *VRN-A1* loci, the Vrn-A1 9K0001 marker originally designed to identify *Vrn-A1a*, was not associated to the predicted spring growth habit in the Spanish landraces collection. Consequently, *Vrn-A1a* allele was also analyzed using the causal marker (VrnA1_k2) previously described in Yan et al. (2004a). The Vrn-A1b-Marq marker effectively distinguished the spring allele *Vrn-A1b*. In addition, the genotypes could be classified as short/long (C/T) or early/late (C/T) according to two SNPs present at *VRN-A1* gene (**Table 1**). Four combinations could be obtained according both SNPs: CC, CT, TC and TT. The CC combination (Claire type; Dıaz et al., 2012) was the most prevalent in the Spanish landraces collection while TC was not present. Considering *vrn-A1*, *Vrn-A1a* and *Vrn-A1b* alleles, and the CC, CT and TT combinations, we could find 5 *VRN* haplotypes in the Spanish collection. Among the genotypes classified as *vrn-A1*, three haplotypes were detected: *vrn-A1_h1 (vrn-A1-TT), vrn-A1_h2 (vrn-A1-CT)* and *vrn-A1_h3 (vrn-A1-CC)*. Both spring alleles *Vrn-A1a* and *Vrn-A1b* were always combined with CC haplotype (*Vrn-A1a_h3 and Vrn-A1b_h3)* (**Figure 2**). Regarding to the modern cultivars, the spring *Vrn-A1b* allele was not detected, and the remaining alleles had a similar percentage to what observed in landraces, except to *vrn-A1_h3* haplotype which showed a reduced frequency in modern cultivars compared to landraces. Respect to *VRN-B1* gene, the most frequent allele was the winter *vrn-B1* allele, although it showed a higher proportion in modern cultivars compared to landraces (**Figure 2**).

We could observe that spring alleles for *VRN-A1* and *VRN-B1* loci were associated with landraces with spring growth habit, only one line classified as winter carried the spring *Vrn-A1b_h3* haplotype, being the winter *vrn-A1* allele present in the remaining 64 winter lines. Among these, 55 lines also had the CC haplotype (*vrn-A1_h3*). Similarly, from 65 Spanish landraces with winter growth habit, 60 carried the winter *vrn-B1* allele and only 1 carried the spring *Vrn-B1a* allele. All modern cultivars with winter growth habit (11) carried the winter *vrn-A1* or *vrn-B1* alleles. On the other hand, among 113 spring landraces, 16 carried the spring *Vrn-A1a_h3* haplotype, 49 carried the spring *Vrn-A1b_h3* haplotype and 69 carried the spring *Vrn-B1a* allele. Of these, 31 had two spring alleles, one at *VRN-A1* and the other at *VRN-B1* locus. Notably, eight landraces classified as spring did not carry any spring allele (**Supplementary Table S1**). In a similar way, from 16 spring modern cultivars, 7 carried the spring *Vrn-A1a_h3* haplotype, and 6 carried the spring *Vrn-B1a*, from which two carried both spring *Vrn-A1a_h3* haplotype and *Vrn-B1a* alleles. In addition, 5 wheat modern cultivars classified as spring did not carry any spring allele (**Supplementary Table S1**).

*PPD-A1* gene did not show polymorphism in this collection as the mutation GS100 (*Ppd-A1a* allele), which confers insensibility to photoperiod, was absent in all the varieties. At the *PPD-B1* locus, the photoperiod-sensitive *ppd-B1* allele was the most prevalent in both landraces and modern cultivars, while the photoperiod-insensitive *Ppd-B1a* allele was rare (detected only in four landraces). At the *PPD-D1* locus, the photoperiod-insensitive allele *Ppd-D1a* was the least frequent in the Spanish landraces, however it was the most common allele in modern cultivars (**Figure 2**), suggesting a selection during breeding programs. On the contrary, the photoperiod-insensitive *Ppd-D1b* allele and the photoperiod-sensitive *ppd-D1* allele were barely represented in modern cultivars. Results indicated that alleles from *PPD-B1* and *PPD-D1* genes were not related to growth habit, as both sensitive and insensitive alleles were randomly distributed across landraces and modern cultivars with either spring or winter growth habit (**Figure 2**).

The *EPS-A1* locus was no polymorphic as the early *Eps-A^m^1e* allele was absent in the collection. At the *EPS-D1* the late *eps-D1* allele was the most frequent in both landraces and modern cultivars, whereas the early *Eps-D1a* allele was found only in 16 landraces and was not represented in modern cultivars. Winter landraces predominantly carried the late *eps-D1* allele, with only 3 out of 65 harboring the early *Eps-D1a* allele. However, the late *eps-D1* allele was also present in 100 out of 113 spring landraces. All modern cultivars carried the late allele regardless of their growth habit (**Figure 2**).

As expected, the haplotype H2 (*Wapo-A1_h2* in this work), which is associated to high SNS, was more represented in modern cultivars than in landraces, in which the haplotype more prevalent was the haplotype H1 (*wapo-A1_h1* in this work) which is associated to low SNS. The haplotype H3 (*wapo-A1_h3* in this work), also associated to low SNS, was either absent or minimally represented in modern and landraces varieties, respectively (**Figure 2**). No relation between *WAPO1* allelic variability and growth habit was observed.

These results suggested that the absence of spring alleles from *VRN-A1* and *VRN-B1* genes is necessary for a winter growth habit. However, other different genes or alleles not characterized in this study must be involved as we could find spring landraces without any identified spring alleles from *VRN-A1* and *VRN-B1*genes in the collection. Yan et al. (2004a) described additional spring alleles that can support this idea. We could not draw conclusions regarding the influence of other genes studied (*PPD1*, *EPS1* and *WAPO1*) on growth habit (in a “winter” background) considering the low number of landraces or modern cultivars (eight and five respectively) that carried *vrn-A1* and *vrn-B1* winter alleles (**Supplementary Table S1**).

### 3.3 Genetic variability for *VRN1, PPD1, EPS1* and *WAPO1* genes in landraces and modern cultivars and relationship to phenology

We aimed to explore the relationship between the genotype and the phenology (thus is DH and DM) in our collection. ANOVA analysis revealed that the genotype and the year had a significant effect in DH and DM across all loci analyzed (p < 0.05). A significant genotype-by-environment (G×E) interaction was observed only for the *VRN-A1* locus in the landraces set (see **Supplementary Table S2**). Consequently, for this case, data were analyzed separately for each year.

#### 3.3.1 Effects of *VRN1* genotype on DH and DM

The significant GxE interaction for the *VRN-A1* gene indicated differential effects on the *VRN-A1* alleles across years in the landraces. In general, DH values were highest on season 2017-2018, which was the coldest and wettest, while the lowest DH values were observed on season 2018-2019, characterized by lower precipitation and higher temperatures (**Supplementary Figure S1**). Similarly, DM values were lower on season 2018–2019 compared to 2020-2021 (**Table 2**).

**Table 2:** Effect of alleles from *VRN-A1* gene on crop development (DH and DM) in Spanish landraces on seasons 2017-2018, 2018-2019, 2020-2021.

Haplotype	Heading day			Maturity day		N
	DH17_18	DH18_19	DH20_21	DM18_19	DM20_21	
*vrn-A1_h1*	181.50 ± 6.98^ab^	145.00 ± 7.45^bc^	167.55 ± 10.53^a^	179.33 ± 7.76^bc^	197.88 ± 7.06^a^	18
*vrn-A1_h2*	184.12 ± 4.58^a^	147.12 ± 6.17^ab^	168.12 ± 6.89^a^	181.87 ± 7.37^ab^	199.12 ± 5.11^a^	8
*vrn-A1_h3*	184.76 ± 5.03^a^	150.22 ± 5.37^a^	172-01 ± 7.73^a^	184.77 ± 6.40^a^	201.66 ± 4.88^a^	85
*Vrn-A1a_h3*	179.75 ± 6.56^b^	142.06 ± 5.45^c^	161.62 ± 9.05^b^	174.93 ± 4.79^c^	194.18 ± 5.51^b^	16
*Vrn-A1b_h3*	183.85 ± 5.93^a^	141.85 ± 5.78^c^	167.45 ± 8.11^a^	176.52 ± 6.56^c^	198.30 ± 5.52^a^	50

DH and DM data are represented as mean ± SD. N indicates the number of varieties carrying specific alleles. Means with the same letter are not significantly different.

To analyze the single effect of *Vrn-A1a* and *Vrn-A1b* alleles on DH and DM, we compared DH and DM among *vrn-A1_h3, Vrn-A1a_h3* and *Vrn-A1b_h3* haplotypes. *Vrn-A1a* and *Vrn-A1b* alleles had been previously described as spring alleles, with an effect in reducing the number of days to heading or to maturity (Yan et al., 2004a). Our results indicated that *Vrn-A1a* had a significant effect on DH and DM in landraces across all seasons, but *Vrn-A1b* only showed significant differences in the 2018–2019 season likely due to the GxE interaction (**Table 2**). Although some of this effect could be attributed to the presence of winter landraces within *vrn-A1_h3* genotype, a significant influence of *Vrn-A1a* or *Vrn-A1b*, depending on the season, was also observed when comparing only spring landraces (**Supplementary Table S3**). In addition, in the modern cultivars, *Vrn-A1a_h3* had a non-significant effect in reducing DH and DM days compared to *vrn-A1_h3* (**Table 3**).

**Table 3:** Allelic effects on DH and DM in Spanish landraces and modern cultivars.

Gene	Allele	Phenotype	Landraces			Modern cultivars		
			N	DH	DM	N	DH	DM
*VRN-A1*	*vrn-A1_h1*	winter	18	164.45 ± 7.96^bc^	188.61 ± 6.94^b^	6	157 ± 5.95^a^	188.75 ± 5.08^a^
	*vrn-A1_h2*	winter/Short	8	166.45 ± 5.23^ab^	190.50 ± 5.92^ab^	6	158.72 ± 1.65^a^	186.83 ± 3.52^ab^
	*vrn-A1_h3*	winter/Short/early	90	169.0 ± 5.50^a^	193.22 ± 4.70^a^	6	153.94 ± 7.87^ab^	185.08 ± 7.16^ab^
	*Vrn-A1a_h3*	Spring/Short/early	16	161.14 ± 6.75^c^	184.56 ± 4.61^c^	7	148.57 ± 7.62^b^	180.92 ± 4.55^b^
	*Vrn-A1b_h3*	Spring/Short/early	55	164.38 ± 5.69^bc^	187.41 ± 5.09b^c^	0	–	–
*VRN-B1*	*vrn-B1*	Winter	111	167.64 ± 6.20^a^	191.24 ± 5.59^a^	20	155.12 ± 7.12^a^	186.03 ± 5.85^a^
	*Vrn-B1a*	Spring	70	164.66 ± 6.34^b^	188.55 ± 6.07^b^	7	151.61 ± 7.67^a^	182.93 ± 5.95^a^
*PPD-B1*	*ppd-B1*	Sensitive	184	166.64 ± 6.36^a^	190.28 ± 5.84^a^	26	153.95 ± 7.30	184.92 ± 5.83
	*Ppd-B1a*	Insensitive	4	158.75 ± 6.08^b^	186.12 ± 9.28^a^	0	–	–
*PPD-D1*	*ppd-D1*	Sensitive	24	165.79 ± 5.23^a^	188.27 ± 4.83^a^	1	157.66^a^	184.5^a^
	*Ppd-D1a*	Insensitive	10	156.20 ± 6.61^b^	182.80 ± 6.15^b^	23	153.50 ± 7.59^a^	184.89 ± 6.20^a^
	*Ppd-D1b*	“Insensitive”	150	167.36 ± 5.81^a^	191.09 ± 5.56^a^	2	157.16 ± 4.94^a^	185.5 ± 1.41^a^
*EPS-D1*	*eps-D1*	Late	172	166.59 ± 6.47^a^	190.30 ± 5.80^a^	28	154.18 ± 7.15	185.07 ± 5.87
	*Eps-D1a*	Early	16	165.20 ± 6.07^a^	189.03 ± 7.23^a^	0	–	–
*WAPO-A1*	*wapo-A1_h1*	Low SNS	153	167.08 ± 6.19^a^	190.75 ± 5.93^a^	8	157.79 ± 7.79^a^	188.56 ± 6.47^a^
	*Wapo-A1_h2*	High SNS	28	162.53 ± 6.30^b^	187.08 ± 5.26^a^	20	152.73 ± 6.52^a^	183.68 ± 5.13^b^
	*wapo-A1_h3*	Low SNS	7	168.95 ± 7.22^a^	190.57 ± 5.05^a^	0	–	–

DH and DM data are represented as mean ± SD. N indicates the number of varieties carrying specific alleles. Means with the same letter are not significantly different.

The short and early C_alleles at *VRN-A1* locus (*h3* haplotype in this study) have been separately described to shorten heading date according to vernalization requirements (Li et al., 2013; Rasheed et al., 2016). However, in our study, the short and early alleles per se did not produce the expected effect on DH and DM under field conditions. In fact, landraces with the longest cycle (highest number of DH and DM) carried the *vrn-A1_h3* haplotype (**Table 2**). The analysis of the mean DH and DM values confirmed these results despite the GxE interaction in landraces but not in modern cultivars (**Table 3**).

At *VRN-B1* locus, the *Vrn-B1a* spring allele showed the expected effect on DH and DM in the collection, reducing the number of days to reach the heading and maturity stages compared to the *vrn-B1* winter allele in both, landraces and modern cultivars (**Table 3**). This effect was related to the growth habit, as was lost when only spring landraces were included in the comparison (**Supplementary Table S4**).

#### 3.3.2 Effects of *PPD1*, *EPS1* and *WAPO1* genotype on DH and DM

The *Ppd-B1a* insensitive allele from *PPD-B1* gene showed an important effect on crop development, reducing the number of days required to reach DH and DM (**Table 3**). However, it is important to note the low frequency of this allele in Spanish landraces and its completely absence in modern cultivars. Similarly, the insensitive *Ppd-D1a* allele significantly decreased DH and DM respect to sensitive *ppd-D1* allele in landraces. (**Table 3**). On the other hand, the insensitive *Ppd-D1b* allele had the opposite effect of what was expected, as landraces carrying this allele needed more days to reach DH and DM stages than those carrying sensitive *ppd-D1* allele (non-significant effect). The influence of alleles from *PPD-D1*gene in the modern cultivars could not be analyzed because of the low variability observed (**Table 3**).

The *EPS1* gene polymorphism analyzed had no effect on DH or DM in the landraces (**Table 3**).

The *Wapo-A1_h2* haplotype, associated with high SNS, caused a DH and DM reduction (5–6 days) compared to *wapo-A1_h1* and *wapo-A1_h3* haplotypes in both landraces and modern cultivars (**Table 3**).

Despite effects on crop development provoked by different alleles, the estimated proportion of the variance indicated that the environment (years) explained a higher percentage of the variation in DH and DM than the different genes both in landraces and modern cultivars, being the year effect higher in DH than DM (**Supplementary Table S5**). On the contrary, the effect of the genes on the variation of crop development was higher in DM than in DH. The gene with higher effect was *VRN-A1*, followed by *PPD-D1*, both in landraces and modern cultivars (**Supplementary Table S5**).

#### 3.3.3 Effect of gene interaction on DH and DM

The gene interaction analysis was conducted on the landraces collection due to the low number of modern cultivars (n=28). ANOVA results indicated that the *VRN-A1* gene interacted with *VRN-B1, PPD-D1* and *EPS-D1* genes while no significant interactions were observed among the remaining genes, except for the genes *VRN-B1* and *WAPO-A1* with respect to DM (see **Supplementary Table S6**).

Due to the interaction showed by *VRN-A1* and *VRN-B1* homologous genes, the effect of alleles from *VRN-A1* gene on DH and DM varied depending on whether the genetic background was *vrn-B1* or *Vrn-B1a* (**Table 4**). The shortest cycles were observed in genotypes with spring *VRN-A1* haplotypes (*Vrn-A1a_h3/vrn-B1, Vrn-A1a_h3/Vrn-B1a* and *Vrn-A1b_h3/Vrn-B1a)* and the *vrn-A1_h1/Vrn-B1a* genotype. *Vrn-B1a* allele did not show a significant effect reducing DH and DM days in *vrn-A1_h2* and *vrn-A1_h3* backgrounds. The longest cycle was associated to the *vrn-A1_h1/vrn-B1* genotype which included 61 of the 65 winter landraces (**Table 4**).

**Table 4:** Gene interaction effects on DH and DM in landraces.

Genotype	DH	DM	N
*vrn-A1_h1/vrn-B1*	169.47 ± 6.88^a^	192.57 ± 5.89^a^	61
*vrn-A1_h2/vrn-B1*	165.13 ± 5.28^abc^	188.70 ± 5.50^ab^	7
*vrn-A1_h3/vrn-B1*	169.03 ± 5.72^a^	193.27 ± 4.89^a^	5
*Vrn-A1a_h3/vrn-B1*	156.44 ± 14.41^d^	181.83 ± 9.22^c^	3
*Vrn-A1b_h3/vrn-B1*	166.18 ± 4.94^abc^	188.61 ± 4.56^ab^	35
*vrn-A1_h1/Vrn-B1a*	161.14 ± 7.28^cd^	186.00 ± 6.81^bc^	26
*vrn-A1_h2/Vrn-B1a*	168.66 ± 5.33^ab^	193.50 ± 6.38^a^	9
*vrn-A1_h3/Vrn-B1a*	169.15 ± 5.14^a^	193.48 ± 4.31^a^	3
*Vrn-A1a_h3/Vrn-B1a*	162.66 ± 3.79^bc^	185.12 ± 3.31^bc^	12
*Vrn-A1b_h3/Vrn-B1a*	160.42 ± 4.39^cd^	184.47 ± 3.95^bc^	19

DH and DM data are represented as mean ± SD. N indicates the number of varieties carrying specific alleles. Means with the same letter are not significantly different.

Respect to homologous *PPD1* genes, we were unable to analyze the effect of different allelic combinations on DH and DM as the *PPD-B1* gene was near monomorphic.

Finally, for a comprehensive perspective, we also examined the interactive effects of the allele combinations from *VRN1/PPD1/EPS1/WAPO1* genes (AC). However, we acknowledge that the complex genetic background and varying sample sizes limit our ability to draw definitive conclusions. In addition, results obtained from landraces may not extrapolate to reference cultivars.

The results identified 45 different AC in the landraces collection, but only 7 were present in more than 4 landraces and were considered for the analysis. The difference in time to reach heading and maturity between the earliest and latest genotypes was 10 and 9 days, respectively. Notably, the *EPS-D1* and *PPD-B1* genes were monomorphic across all seven ACs, preventing their effects from being analyzed.

Although the *Wapo-A1_h2* haplotype was only present in one of the seven AC, a comparison between AC5 and AC6, suggests that *Wapo-A1_h2* has no effect in a spring genetic background (**Table 5**).

**Table 5:** Different allele combinations in landraces varieties are showed.

AC	Allelic combination	DH	DM	N
1	*vrn-A1_h3:vrn-B1:ppd-B1:Ppd-D1b:eps-D1:wapo-A1_h1*	170.26 ± 4.23^a^	194.01 ± 4.16^a^	50
2	*vrn-A1_h3:Vrn-B1a:ppd-B1:Ppd-D1b:eps-D1:wapo-A1_h1*	169.05 ± 3.21^a^	193.82 ± 3.26^a^	17
3	*Vrn-A1b_h3:vrn-B1:ppd-B1:Ppd-D1b:eps-D1:wapo-A1_h1*	166.60 ± 4.58^ab^	188.95 ± 4.44^b^	33
4	*vrn-A1_h1:Vrn-B1a:ppd-B1:Ppd-D1b:eps-D1:wapo-A1_h1*	164.05 ± 4.39^bc^	188.66 ± 1.47^b^	6
5	*Vrn-A1a_h3:Vrn-B1a:ppd-B1:ppd-D1:eps-D1:Wapo-A1_h2*	163.75 ± 0.41^bc^	186.75 ± 1.32^b^	4
6	*Vrn-A1a_h3:Vrn-B1a:ppd-B1:ppd-D1:eps-D1:wapo-A1_h1*	163.33 ± 4.32^bc^	185.5 ± 3.80^b^	5
7	*Vrn-A1b_h3:Vrn-B1a:ppd-B1:Ppd-D1b:eps-D1:wapo-A1_h1*	160.63 ± 4.63^c^	185.12 ± 3.81^b^	12

Data from DH and DM are means ± SD. Means with the same letter are not significantly different. N indicates the number of varieties bore of each specific combination.

Regarding *PPD-D1* gene, the insensitive *Ppd-D1a* allele was absent and the insensitive *Ppd-D1b* allele was mainly represented in the late-maturing ACs (1, 2, 3 and 4), behaving as a sensible allele, increasing the number of days to heading and maturity, like previously observed in the section 2.3.1. On the other hand, the presence of two spring alleles from *VRN1* significantly reduced the number of days to heading and maturity (AC5-AC7), whereas genotypes without spring alleles required more time to reach these stages. Furthermore, the *h3* haplotype appeared to counteract the cycle-shortening effect of a single spring allele from *VRN1* genes (AC1, AC2 and AC4). However, when two spring alleles from *VRN1* genes were present, they overrode the effect of *h3* haplotype (AC5, AC6, and AC7), leading to shorter DH and DM (**Table 5**).

There is to highlight that we did not dispose of all allelic combinations. Likely, an allelic combination comprising all spring/early alleles together (*Vrn-A1a_h3:Vrn-B1a:Ppd-B1a:Ppd-D1a:Wapo-A1_h2*) would result in earlier flowering than any other allelic combination considered in this study.

## 4 Discussion

The replacement of local landraces by high-yielding wheat varieties that began with the Green Revolution has led to a loss of genetic variation in wheat varieties. This depletion has now encouraged the use of genetic resources (such us landraces, wild relatives and isolated breeding gene pools) in wheat breeding programs significantly impacting the crop (King et al., 2024). With this aim, the genetic variability of a bread wheat collection of Spanish landraces was characterized at targeted loci, showing an outstanding degree of diversity, and representing a valuable genetic resource useful for exploitation in breeding programs (Pascual et al., 2020a). However, the variability existing in Spanish bread wheats for vernalization, and photoperiod genes had been poorly analyzed. In the present study, we used molecular markers to assess the allelic variation at the *VRN-A1, VRN-B1, PPD-A1, PPD-B1, PPD-D1, EPS-A1, EPS-D1* and *WAPO-A1* loci in a collection of Spanish bread landraces, also using a set of modern cultivars for comparison. We examined the effect of individual alleles and allelic combinations on growth habit and phenology in modern cultivars and landraces under field conditions during three crop seasons. The use of germplasm pools (landraces growth in Spain and modern cultivars) allowed us to test the correspondence between ancient and recent genetic backgrounds regarding growth habit and the variation in crop development.

### 4.1 Breeding effect on crop development

Bread wheat is generally classified as spring or winter type according to its vernalization requirements for a proper flowering time (Flood and Halloran, 1984; Trevaskis et al., 2007; Kolev et al., 2010). Winter wheat types are ancestral to spring wheat types (Flood and Halloran, 1986) and the insensitivity of spring wheat to vernalization is responsible for its early flowering ability (Pugsley, 1971). In our study, among 59.3% of modern cultivars and 63.5% of Spanish landraces presented a spring growth habit compared with 36.5% and 40.7% showed a winter growth habit and as expected, spring varieties were earlier than winter varieties. The difference in proportions between cultivars or Landraces was not significant (data not shown). This reflects the diverse climatic conditions in Spain, which fostered adaptation to both spring and winter wheat types.

The comparison of DH and DM in the collection of wheat landraces and the set of modern cultivars showed that breeding efforts have significantly shortened the crop cycle. This reduction was observed in both DH and DM, consistent with previous studies reporting a shortening of around 2 and 9 days in the time to anthesis, in Italian and Spanish durum wheat varieties, respectively, because of breeding activities throughout the 20th century (Álvaro et al., 2008; Royo et al., 2008; Isidro et al., 2011; Royo et al., 2020). The selection for earlier flowering varieties has been a key objective of breeding programs, especially in regions like Spain, where increasing drought and temperature stress in the spring make early grain filling essential for optimal yields (Royo et al., 2006). There is to highlight that the modern cultivars needed fewer days to reach heading and maturity than Spanish landraces regardless of the allelic combinations at *VRN1, PPD1* and *WAPO1*. This suggests that breeding programs have not only selected for specific alleles linked to earliness but have likely targeted other alleles influencing developmental timing as well.

### 4.2 Genetic variability for *VRN1, PPD1, EPS1* and *WAPO1* genes in landraces and modern cultivars and relationship to growth habit

At *VRNA1* locus, the *Vrn-A1a* and *Vrn-A1b* alleles have been described as spring-type alleles (Yan et al., 2004a), although the *Vrn-A1b* allele is not always associated with spring growth habits in tetraploid and hexaploid lines (Pidal et al., 2009; Shcherban et al., 2015; Strejcková et al., 2021). In our study, from the 65 winter landraces, only one carried the *Vrn-A1b* spring allele and another one carried the spring *Vrn-B1a* allele. These results suggest again the key role of *VRN1* alleles in wheat growth habit. Our results have shown that both spring alleles, *Vrn-A1a* and *Vrn-A1b*, are combined with *h3* haplotype (C-C haplotype) in the Spanish collection. Similarly, Chen et al. (2011) also reported that cultivars carrying the dominant spring *Vrn-A1a* polymorphism always exhibit the C allele in exon 7 of *VRN-A1*. However, they also reported that those genotypes carrying the recessive winter *vrn-A1* allele always exhibit the T allele, proposing the C/T polymorphism as a good predictor of spring/winter growth habit (Chen et al., 2011). However, our results do not support this hypothesis, as we found varieties with *vrn-A1* allele harboring either C or T allele in exon 7. Similar findings were reported by Makhoul et al. (2022), which showed that 6 out of 11 cultivars carrying the C allele had the recessive *vrn-A1* allele, while the other five cultivars had the dominant *Vrn-A1a* allele. Muterko and Salina (2018) also found that while most, but not all, of wheat genotypes carrying the recessive *vrn-A1* allele have a T allele in exon 7, some dominant *Vrn-A1* alleles also carried it. All these results suggest that the C/T polymorphism in exon 7 is not a reliable predictor of spring/winter type growth habit as previously suggested (Chen et al., 2011).

Thirteen spring lines, including modern cultivars and landraces, did not present any spring alleles from *VRN-A1* and *VRN-B1* genes. Despite the fact that alleles from the *VRN-D1* gene have not been tested in the complete Spanish collection, we have proved that the spring *Vrn-D1a* allele (Fu et al., 2005) is only present in seven of thirteen spring lines (data don’t show), which do not contain any spring alleles in the *VRN-A1* and *VRN-B1* genes, suggesting that other alleles (not tested in this work) could be implicated in regulation of growth habit in this collection. It is known that the most common source of spring growth habit is a dominant mutation at one or more *VRN1* loci (*VRN-A1, VRN-B1, VRN-D1*) (Trevaskis, 2010; Yan et al., 2004a). However, given that alleles from *VRN-D4* (Kippes et al., 2015), *VRN3* (Yan et al., 2006), *VRN2* genes (Zhu et al., 2011) and other spring *VRN-1* alleles (review in Milec et al., 2023 and Afshari-Behbahanizadeh et al., 2024) did not analyze in this project can also influence flowering time and growth habit, future investigations into these loci could offer further insights into the molecular basis of growth habit in Spanish wheat landraces.

On the contrary to what was observed with alleles from *VRN1* genes, and as expected, different alleles from *PPD1, EPS1* and *WAPO-A1* did not have any effect on growth habit (**Figure 2**).

### 4.3 Genetic variability for *VRN1, PPD1, EPS1* and *WAPO1* genes in landraces and modern cultivars and relationship to phenology

*VRN1, PPD1*, and *EPS1* genes play critical roles in regulating phenology and adaptation to specific environmental conditions, making them important for breeding programs aiming to optimize yields.

It is known that the *VRN-A1* gene has a notably impact on DH and DM. Using a Vrn-A1_9K0001 KASP marker for the detection of *Vrn-A1a* allele, their influence on DH and DM did not agree with those previously described in other studies (Royo et al., 2020). To check this disagreement, we performed an additional screening by a VrnA1_k2 PCR marker (Yan et al., 2004a) and we proved that the KASP assay Vrn-A1_9K0001 that presumably distinguishes winter (*vrn-A1*) and spring type *Vrn-A1a* allele (Rasheed et al., 2016) did not work properly in our Spanish collection. This discrepancy might be due to potential recombination events between the *VRN-A1* gene and the Vrn-A1_9K0001 marker, suggesting that the marker may not be an accurate tool for assessing allelic variation of *VRN-A1* gene in some landrace collections. This finding emphasizes the need for careful marker selection in breeding programs to avoid incorrect evaluations.

In this study, we found that the spring *Vrn-A1a* allele was only present in a quarter of the modern cultivars, while the spring *Vrn-A1b* allele was entirely absent. This absence is consistent with reports from global wheat collections, such a 276 ICARDA collection and 2,529 CIMMYT genotypes, where *Vrn-A1b* was either absent or rarely observed (Review in Royo et al., 2020), confirming its elimination from the programs of two of the most important germplasm providers worldwide. Moreover, spring *Vrn-A1b* allele was near absent in fifty-nine Pakistani wheat cultivars, which probably proceeded from CIMMYT material (Iqbal et al., 2011), and in a collection of breeding materials from the winter wheat gene bank of the Agricultural Institute from Hungary (Kiss et al., 2014). Similarly, only eight and one cultivars from a total of 278 Chinese wheat cultivars (Zhang et al., 2008) and 134 recent Japanese modern cultivars (Mizuno et al., 2022), respectively, carried the *Vrn-A1b* allele. On the other hand, the spring *Vrn-A1b* allele was the most frequent spring allele in Spanish landraces. *Vrn-A1b* allele was associated with a shorter development time, though to a lesser extent than the *Vrn-A1a* allele (**Table 2**). These results are in line with those reported in durum wheat by Royo et al. (2020), who found that the *Vrn-A1b* allele was associated with early heading time. The reason for the exclusion of *Vrn-A1b* in modern germplasm remains unclear and cannot be attributed solely to its impact on phenology. Selection of the spring allele *Vrn-A1a* in breeding programs could be justified by its major effect on crop development, although this only could be observed in landraces. In addition, both spring alleles, *Vrn-A1a* and *Vrn-A1b*, are always combined with *h3* haplotype, which have been described to have an opposing effect on crop development (Rasheed et al., 2016; Li et al., 2013; Dıaz et al., 2012). The *h3* haplotype presence provoked that the effect on crop development from *Vrn-A1b* allele (haplotype *Vrn-A1b_h3)* could not be observed respect to wild type *vrn-A1_h1* haplotype (**Table 2**). It is possible that if *Vrn-A1b* allele is always combined with *h3* haplotype its effect on crop development is always masked, preventing its selection. In fact, previous works described that the two *VRN-A1* SNPs analyzed separately, short C_allele (Jagger-type) and early C_allele (Claire –type), caused a decrease in DH and DM (Rasheed et al., 2016; Li et al., 2013; Dıaz et al., 2012), although such alleles were not predominant in the panel studied. It is noteworthy that, to our knowledge, the effect of these haplotypes in combination with *Vrn-A1a* and *Vrn-A1b* alelles had not been addressed. On the contrary, in our field conditions, the *h3* haplotype (in a *vrn-A1* background) increased DH and DM in Spanish landraces but caused a non-significant reduction in growth cycle in modern cultivars. This suggests that the genetic background of the landraces may modulate the effects of these alleles, underscoring the importance of considering genetic background when selecting for flowering time traits in breeding programs.

Similar to the spring alleles from *VRN-A1*, the *Vrn-B1a* allele, present in approximately 38% of our landraces and 25% of modern cultivars (**Supplementary Table S1**), was associated with a spring growth habit. The *Vrn-B1a* allele has also been shown to reduce developmental time in other wheat collections, being predominantly found in bread wheat but rare in durum landraces (Royo et al., 2020; Maccaferri et al., 2019; Mizuno et al., 2022; Zhang et al., 2008; Kiss et al., 2014; Rasheed et al., 2016). However, our results did not align with these previous findings, as the *Vrn-B1a* allele did not show an effect on DH and DM in either landraces (**Supplementary Table S4**) or modern cultivars (**Table 3**). Nevertheless, based on the clear association of *Vrn-B1a* allele with a spring growth habit, along with its link to shortened developmental time in the Spanish collection, we propose that *Vrn-B1a* could be a valuable allele for future breeding programs, particularly in regions with diverse climatic conditions such as Spain.

The combination of alleles from different *VRN1* genes in this study showed that landraces carrying fewer spring alleles (*Vrn-A1a, Vrn-A1b*, and *Vrn-B1a*) required more days to reach heading and maturity. Furthermore, spring *Vrn-A1* alleles (a and b) were found to have a stronger effect on phenology than spring *Vrn-B1* alleles, with their effects being additive consistent with other studies (Stelmakh, 1992; Zhang et al., 2008). These results underscore the significant role of spring alleles from *VRN1* genes in reducing the number of days to heading and maturity, contributing not only to a spring growth habit but also to faster flowering.

In terms of the photoperiod pathway, the photoperiod-insensitive *Ppd-B1a* allele, despite its high effect on development observed in Spanish landraces, does not seem to have been prioritized in breeding programs, as it was barely present and did not cause any effect on DH in the modern cultivars. The early effect on crop development of *Ppd-B1a* allele has been also previously described (Dıaz et al., 2012; Mizuno et al., 2022; Kiss et al., 2014) although, in a similar way to our studies, in all these studies the *Ppd-B1a* allele was underrepresented. The *Ppd-D1a* allele, which is photoperiod-insensitive and often selected for earliness, was highly represented in modern cultivars, further confirming the importance of this allele in breeding programs. Similarly, this allele is highly represented in a cultivar panel analyzed by Rasheed et al. (2016), and in most of the cultivars analyzed in diverse collections (Mizuno et al., 2022; Iqbal et al., 2011; Kiss et al., 2014). Breeding selection is reasonable considering the effect that the insensitive *Ppd-D1a* allele have on crop development in landraces. No significant effects on crop development were observed in the modern cultivars, likely as a consequence of the low number of varieties analyzed. On the other hand, the *Ppd-D1b* allele is very common in Spanish landraces and underrepresented in modern cultivars (**Figure 2**), due probably to its little effect on crop development.

The insensitive *Ppd-D1a* allele reduced DH and DM, whereas the *Ppd-D1b* allele unexpectedly delayed these stages despite being also classified as an “insensitive” allele (Rasheed et al., 2016). However, this antagonistic effect has been previously observed in barley (Fernández-Calleja et al., 2021; Slafer et al., 2023), where photoperiod-sensitive allele from *PPD-H1* conferred earliness in barley while in wheat, genotypes carrying the photoperiod-insensitive allele where those that flowered earlier. The discrepancy between wheat and barley is attributed to the different polymorphism in these species: in wheat, the insensitive alleles result from transposon insertion or deletions in the promoter (like *Ppd-D1a* allele) or copy number variations (like *Ppd-B1a*), while in barley the polymorphisms affect the coding region (CCT domain) (review in Fernández-Calleja et al., 2021 and in Slafer et al., 2023). The polymorphism identified in this study with the TaPpdDD002 marker reveals a 5 bp deletion in exon 7 that generates a stop codon upstream of the conserved CCT domain (Beales et al., 2007). This suggests that the “insensitive” *Ppd-D1b* allele in wheat is molecularly like the sensitive alleles in barley. Sequencing of the region in landraces carrying the *Ppd-D1b* allele confirmed the presence of the same 5 bp deletion as described by Beales et al. (2007). The CCT (CONSTANS, CO-like, and TOC1) domain is usually described as a highly conserved region located near the C-terminus of plant proteins involved in light signal transduction. CCT domain-containing proteins have been described to interact with both the Evening Complex in Arabidopsis (Huang et al., 2016) and the Florigen Activation Complex in rice (Cai et al., 2021). Recently, Fourquet et al. (2024) suggested a potential molecular interaction in wheat between PPD-D1 and members of the Evening Complex (ELF3-D and FT1-B), via CCT domain. Additionally, Li et al. (2024) also indicated that the genetic interaction between photoperiod and the floral pathway gene *GIGANTEA* occurs only in the presence of the photoperiod-sensitive *Ppd-D1b* allele. These results provide evidence of the critical role of interactions between floral pathway genes and *PPD-D1*, likely via CCT domain.

This *Ppd-D1b* allele was first described in the photoperiod sensitive variety ‘Norstar’ (Beales et al., 2007) and was screened in a panel comprising 300 diverse cultivars from China and 13 other countries, in which *Ppd-D1b* allele provoked a non-significant delay in DH and DM of 2 days (Rasheed et al., 2016), something similar to our observations. Additionally, studies in the ‘Paragon’ genetic background demonstrated that the delay effect of the ‘Norstar’ allele was only apparent when *Ppd-B1a* was absent, suggesting that the *Ppd-D1b* allele’s effects might be mitigated by functional *PPD1* alleles on other chromosomes (Shaw et al., 2013). Our collection included only four landraces with the *Ppd-B1a* allele (despite high effect on developmental time in Spanish landraces), which likely prevent the buffer effect from *PPD-B1* gene described by Shaw et al. (2013). Consequently, the “insensitive” *Ppd-D1b* allele extended DH and DM in our Spanish collection. This allele was unlikely selected in breeding programs, unlike the *Ppd-D1a* allele. It is important to note that these results were obtained under field conditions, where photoperiod was not controlled. Future studies with controlled photoperiod conditions may provide more insight into these interactions.

The 9–10 day difference in heading observed in this study due to the interaction of vernalization and photoperiod genes, aligns with previous findings in bread wheat (Kiss et al., 2014) and durum wheat (Royo et al., 2020), where similar differences in flowering time were reported (10 and 11 days, respectively).

*EPS1* genes regulate residual variations in flowering time between genotypes once vernalization and photoperiod requirements are covered (Prieto et al., 2018; Snape et al., 2001; Slafer, 1996). Although *EPS-D1* has been described to regulate flowering (Wittern et al., 2023), the early *Eps-D1a* allele was not present in Spanish modern cultivars, probably due their limited effect on developmental time as depending on VRN and PPD genotypes. Jardón et al. (2023) found that, on average, lines carrying the “early” *EPS-D1* allele (*Eps-D1a* allele in this work) headed earlier under controlled vernalization and photoperiod conditions, highlighting the role of this gene in flowering regulation. However, the *Eps-D1a* allele did not appear to influence developmental stages in our Spanish landrace collection in our Mediterranean field conditions.

The *WAPO-A1* gene has been described to regulate SNS in durum and bread wheat (Wittern et al., 2022). SNS can also been influenced by genes affecting flowering time through vernalization, photoperiod or earliness per se pathways: *VRN1, FUL2, FUL3, PPD1*, *VRN3/FT1, ELF3* (Li et al., 2019; Shaw et al., 2013; Brassac et al., 2021; Alvarez et al., 2016). However, *WAPO-A1* native alleles have not been reported to cause large changes in flowering time in field trials (Muqaddasi et al., 2019; Katz et al., 2022) and, while transgenic expression delayed flowering, CRISPR and EMS mutants exhibited no significant effects (Kuzay et al., 2022). In contrast, our results suggested a potential role of *WAPO-A1* gene regulating DH and DM in both modern cultivars and landraces varieties. Previous correlation studies between flowering time and number of spikelets detected, on the one hand, a significant correlation for total number of spikelets with heading and flowering periods (Katz et al., 2022) and, on the other hand, a negative correlation between DH/DM and SNS (López-Fernández et al., 2023). Our results suggest that the high SNS might be a consequence of early flowering caused by *Wapo-A1_h2* haplotype. According to this result, *Wapo-A1_h2* haplotype, selected in breeding programs by its role in SNS, might also contribute to the regulation of flowering.

## 5 Conclusion

Our findings highlight the efforts made in wheat breeding programs to optimize crop development, particularly with respect to earliness, and demonstrate the valuable genetic diversity still present in Spanish wheat landraces. While breeding programs have successfully reduced the time to heading and maturity, there remains underutilized genetic variability that could be harnessed for more precise regulation of flowering time and better adaptation to diverse environmental conditions. The allelic diversity identified in Spanish landraces, especially in *VRN1*, *PPD1*, and *WAPO-A1*, presents promising avenues for future wheat improvement, particularly in regions with complex and changing agro-climatic conditions.

## Data Availability

The original contributions presented in the study are included in the article/**Supplementary Material**. Further inquiries can be directed to the corresponding author.
