# Supplementary material for: Breeding potential of Spanish bread wheat landraces: genetic variability in vernalization and photoperiod sensitivity
Source: Front Plant Sci. 2025 Oct 30;16:1593667. doi: 10.3389/fpls.2025.1593667 (PMC12611846; doi:10.3389/fpls.2025.1593667)
Supplement: Supplementary file 1 [file DataSheet1.pdf]

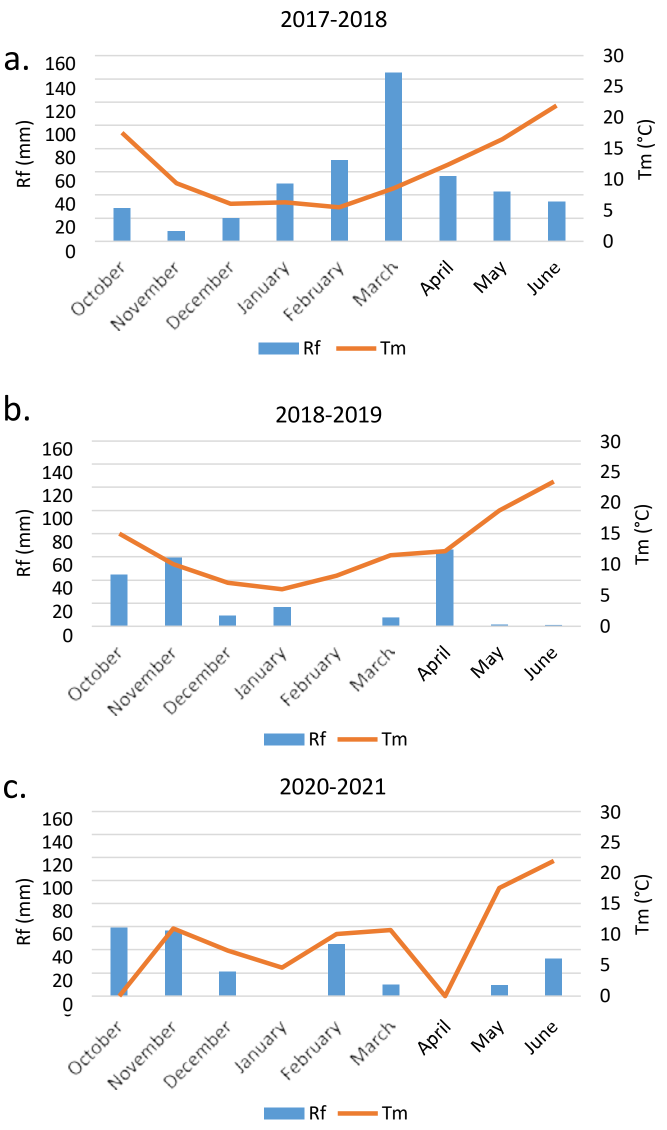

Supplementary Figure 1. Monthly rainfall (mm), in blue, and mean temperature (Tm, ºC), in orange, recorded from October to June at the field trial sites during (A) 2017-2018, (B) 2018-2019 and (C) 2020-2021. Due to errors at the weather station, rainfall data are missing for January and April 2020, and temperature data are missing for October 2020 and April 2021.
